# Source Attribution of Foodborne Diseases: Potentialities, Hurdles, and Future Expectations

**DOI:** 10.3389/fmicb.2018.01983

**Published:** 2018-09-03

**Authors:** Lapo Mughini-Gras, Pauline Kooh, Jean-Christophe Augustin, Julie David, Philippe Fravalo, Laurent Guillier, Nathalie Jourdan-Da-Silva, Anne Thébault, Moez Sanaa, Laurence Watier, The Anses Working Group on Source Attribution of Foodborne Diseases

**Affiliations:** ^1^Centre for Infectious Disease Control (CIb), National Institute for Public Health and the Environment (RIVM), Bilthoven, Netherlands; ^2^Faculty of Veterinary Medicine, Utrecht University, Utrecht, Netherlands; ^3^Risk Assessment Department, French Agency for Food, Environmental and Occupational Health & Safety (Anses), Maisons-Alfort, France; ^4^Ecole Nationale Vétérinaire d'Alfort, Maisons-Alfort, France; ^5^Ploufragan-Plouzané Laboratory, French Agency for Food, Environmental and Occupational Health & Safety (Anses), Ploufragan, France; ^6^NSERC Industrial Research Chair in Meat-Safety (CRSV), Faculty of Veterinary Medicine, University of Montreal, Saint-Hyacinthe, QC, Canada; ^7^Laboratory for Food Safety, French Agency for Food, Environmental and Occupational Health & Safety (Anses), Maisons-Alfort, France; ^8^The French National Public Health Agency, Saint-Maurice, France; ^9^Biostatistics, Biomathematics, Pharmacoepidemiology and Infectious Diseases (B2PHI), Inserm, UVSQ, Institut Pasteur, Université Paris-Saclay, Paris, France

**Keywords:** source attribution, foodborne pathogen, mathematical modeling, molecular typing, population genetics, phenotyping, foodborne disease, frequency-matching models

Attributing human cases of foodborne diseases to putative sources of infection is crucial for identifying targets for interventions in the food production chain (Pires et al., 2009). Beyond traditional epidemiological approaches to source attribution, including outbreak investigations (Pires et al., 2010) and case-control studies of sporadic cases (Fullerton et al., 2012), which are undermined by several factors like simultaneous exposure to multiple sources and selection/information bias, a number of microbiological approaches have been developed (Pires et al., 2014). These approaches are based on statistical modeling of microbial subtyping data, which often derive from integrated surveillance systems of human cases and pathogen occurrences in selected animal, food, and environmental sources (Pires et al., 2009).

The so-called “frequency-matching” source attribution models, such as the (modified) Dutch (van Pelt et al., 1999; Mughini-Gras et al., 2014b) and Danish (“Hald”) models (Hald et al., 2004; Mullner et al., 2009a; David et al., 2013a), which rely on the one-to-one matching of microbial subtypes in humans and sources, have been extensively used for source attribution of major (bacterial) foodborne pathogens. Studies have focused on *Salmonella* (Hald et al., 2004, 2007; Mullner et al., 2009a; Guo et al., 2011; David et al., 2013b; Mughini-Gras et al., 2014a,c; De Knegt et al., 2015; de Knegt et al., 2016; Vieira et al., 2016) and *Campylobacter* (Mullner et al., 2009a,b; Boysen et al., 2014), and to a lesser extent on *Listeria* (Little et al., 2010; Nielsen et al., 2017) and Shiga-toxin producing *E. coli* (STEC) (Mughini-Gras et al., 2018b). In these studies, subtypes were defined by either phenotyping (e.g., serotyping, phage-typing, antimicrobial resistance) or genotyping (e.g., multi-locus sequence typing and multi-locus variable number tandem repeat analysis). Other source attribution approaches are based on the genetic relatedness among isolates from humans and sources. These are population genetics models like STRUCTURE (Pritchard et al., 2000) and the asymmetric island model (Wilson et al., 2008), which have different genetic targets (e.g., allele numbers, microsatellites, single nucleotide polymorphisms). Applications of these models are mainly limited to *Campylobacter* (Wilson et al., 2008; Sheppard et al., 2009; Strachan et al., 2009, 2012; Mughini Gras et al., 2012; Smid et al., 2013; Mossong et al., 2016) and, to a far lesser extent, *Salmonella* (Mughini-Gras et al., 2014c; Barco et al., 2015), and *Listeria* (Nielsen et al., 2017). The scope of pathogens addressable with these models will increase in the years to come as the Whole Genome Sequencing (WGS) “revolution” is increasing the acquisition of high-throughput data (Franz et al., 2016). While the high discriminatory power of molecular data can be adjusted to use the frequency-matching models (de Knegt et al., 2016), population genetics approaches are a more promising way forward. They namely allow assessing the genealogical history and evolutionary relationships among strains, taking into account mutation, recombination and migration events. A characteristic of population genetics models is that strain types found exclusively in humans and not in sources can still be attributed, usually to the genetically closest. This can be considered as either an advantage or a limitation. Whatever the case may be, it is of value when perfect matches of strain types between humans and sources are unattainable given the highly discriminatory genetic targets investigated and the usually limited number of sources represented. For WGS-based source attribution, it is essential to rely on networks of laboratories that share genomic (and epidemiological) data, working altogether toward harmonizing methods, inputs, and outputs.

For selected pathogens for which data on the level of contamination along the food production chain, consumer's practices and dose-response relationships are available, quantitative risk assessment (QRA) offers another option for “bottom-up” source attribution. QRA models have the potential to estimate the proportion of cases attributable to sources for all points in the food production chain, accounting for factors (e.g., food processing, storage, consumption) that are otherwise difficult to address with typical disease surveillance tools. Yet, incorporating strain virulence variation in dose-response models remains challenging.

Epidemiological and microbiological approaches, however, are not mutually exclusive and can be combined in a “source-assigned case-control study” design, as illustrated for *Campylobacter* (Mullner et al., 2010; Bessell et al., 2012; Mughini Gras et al., 2012; Lévesque et al., 2013; Mossong et al., 2016; Rosner et al., 2017), *Salmonella* (Mughini-Gras et al., 2014b), and STEC (Mughini-Gras et al., 2018b). Source attribution at the point of exposure is also possible by combining comparative exposure assessment and subtype comparison based on comparative genomic fingerprinting (Ravel et al., 2017). These combined analyses show that the outcome of epidemiological studies and QRA can be enhanced by incorporating source attribution and vice versa.

The choice of the source attribution method depends on the point of attribution along the farm-to-fork continuum and the epidemiological context, as well as the quality/completeness of data available and the characteristics of the pathogen in question. For microbiological methods, the sampling point of the sources determines the point of attribution, hence the risk management target (Mughini-Gras et al., 2014a). Indeed, while epidemiological methods like case-control studies are best suited to attribute sporadic cases “downstream” to specific food exposures, transmission routes and risk factors, microbiological methods can attribute sporadic cases up to the level of reservoir (i.e., amplifying host). Moreover, when using frequency-matching models, the subtypes upon which attribution relies must possess some stability along the farm-to-fork continuum, as they are often compared between primary production and human cases. If this is not the case, population genetics models, which account for evolution, are to be preferred. Microbiological methods, however, are not suited to attribute pathogens with low genotyping/phenotyping diversity (i.e., non-heterogeneous distribution among sources), and pathogens with only one recognized reservoir in a given setting (e.g., cats for *Toxoplasma gondii*). Moreover, QRA models can be used to assess the relative contributions of different reservoirs, transmission routes, risk factors and exposures, but in practice they are mainly used to assess a limited number of sources to which consumers are directly exposed.

Data availability and quality are major factors guiding the choice of source attribution methods, and doubtlessly the relevance of results. Inferring probabilistically the most likely sources of human cases based on subtyping data is demanding in terms of data requirements and computational capacity. Besides large strain-typed data sets for a broad panel of potential sources representative of the epidemiological situation in question, it may be necessary to include other data on, e.g., the level of contamination of the sources and food consumption. Therefore, comprehensive application of microbiological approaches to source attribution are often conditional to well-established surveillance systems, detailed ancillary data to frame the specific epidemiological context, and the systematic and harmonized application of subtyping methods. This requires including source attribution as an objective of surveillance systems to generate data optimized for this purpose. Yet, studies providing indications on the optimal sample size to address both statistical power and strain diversity in source attribution are scarce (Smid et al., 2013).

The relevance of the attributions based on microbiological methods depends also on the number of sources considered, which need to be as complete and representative as possible. Omission of epidemiologically-relevant sources can seriously affect the attributions. A characteristic of the frequency-matching models is that they do not allow attributing subtypes identified only in humans, thereby generating a fraction of non-attributable cases. Yet, human cases related to sources that are not included in the model, but are infected with subtypes present also in other sources included in the model, will be erroneously attributed to these latter sources. It has been shown that including sources considered of minor importance led to the reassignment of 25% of the cases initially attributed to known sources of *Salmonella* (David et al., 2013b). The panel of potential sources included in the analysis is particularly important for population genetics models, as no non-attributable fraction is contemplated. Non-omission of relevant sources is also relevant when there are important non-foodborne sources, as illustrated for environment-mediated spread of *Campylobacter* (Friesema et al., 2012; Mughini-Gras et al., 2016b) and pet-associated salmonellosis (Mughini-Gras et al., 2016a) and campylobacteriosis (Mughini Gras et al., 2013). However, such non-food sources are not usually monitored by surveillance systems. Moreover, source attribution based on microbial subtyping often suffers from incomplete time series for all relevant sources. This makes it difficult to assess trends in the relative contributions of different sources and to document the impact of interventions, even if methods attempting to address this have been proposed (Ranta et al., 2011).

Accounting for biological characteristics of the different strains remains challenging for models based on microbiological methods, as well as QRA. Moreover, these models often assume that the different strains and sources are independent entities, whereas biological interactions between these exist. Recently, a new source attribution model, named sourceR, has been proposed (Miller et al., 2017). This model builds upon, and blends together, properties of the original (Hald et al., 2004) and modified (Mullner et al., 2009a) Hald models, using molecular surveillance data to determine the force of infection from each source, allowing for varying survivability/pathogenicity of strains and varying abilities of sources to act as vehicles (Miller et al., 2017). A Bayesian non-parametric approach is used to cluster strains by epidemiological behavior, preventing the model from overfitting and allowing for the detection of “highly virulent” strains (Miller et al., 2017). This is a significant improvement over previous models that relied on several adaptations to improve identifiability, such as fixing some parameters *a priori* (Hald et al., 2004), modeling them hierarchically as random effects (Mullner et al., 2009a), or setting the ones related to a unique source using data-based values (David et al., 2013a). Future developments will have to focus on modeling also spatiotemporal effects to identify, e.g., foci of source contamination in time and space, particularly for pathogens that rapidly evolve over time and are highly diverse across regions (Miller et al., 2017). Moreover, including interactions between types and sources would allow for certain types to be differentially likely to cause disease depending on the source they appear in (Miller et al., 2017). There is also a need to account for multi-directionality in transmission from and to the sources and within the human population itself, as this is a limiting factor for source attribution of microorganisms for which humans can be both targets and sources. This could be the case of, e.g., *Shigella, Cryptosporidium*, or Extended-Spectrum Beta-Lactamase (ESBL)-producing bacteria.

The development of new source attribution models should go in parallel with the development of model diagnostics and empirical cross-validation tools, similar to traditional validation based on self-attribution (Sheppard et al., 2009; Kittl et al., 2013; Mughini-Gras et al., 2014c). In addition, difficulties in identifying a model that satisfies all needs should lead to the routine application of different models in a comparative fashion, including indexes of genetic proximity and diversity (e.g., Simpson's index of diversity and analysis of molecular variance) (Excoffier et al., 2005; Sheppard et al., 2009; Kittl et al., 2013; Roux et al., 2013), and phylogenetic approaches (Didelot and Falush, 2007; Strachan et al., 2009; Roux et al., 2013; Mughini-Gras et al., 2014c) to complement source attribution (Dearlove et al., 2016). Comparison with epidemiological approaches (Roux et al., 2013) or different population genetics models like BAPS (Dale et al., 2011) are also available. Obviously, using new models requires evaluating changes in trends of attributions as to check whether they reflect actual changes in the epidemiology of these pathogens or artifacts of the different methods used (Mughini-Gras et al., 2018a).

In conclusion, with increased interest in source attribution of foodborne pathogens, current methods need to be systematically sorted and possibly combined or applied in a comparative fashion, accounting for factors like the type, quality, and completeness of data available, analytical requirements, point of attribution, pathogen characteristics, and epidemiological contexts. As this field evolves, a number of methodological bottlenecks will have to be faced, including the analysis of increasingly available high-throughput data, spatiotemporal and multi-directional processes, and the yet to be determined differential properties (pathogenicity and behavior) of pathogen strains in interaction with the sources.

## Author contributions

This opinion resulted from experts' discussions at the ANSES' working group on Source Attribution of Foodborne Diseases, which was coordinated by PK. LW and MS supervised the project. LM-G wrote the manuscript draft with input from all other authors.

### Conflict of interest statement

The authors declare that the research was conducted in the absence of any commercial or financial relationships that could be construed as a potential conflict of interest.
